# Association of DENND1A Gene Polymorphisms with Polycystic Ovary Syndrome: A Meta-Analysis

**DOI:** 10.4274/jcrpe.2259

**Published:** 2016-06-06

**Authors:** Shan Bao, Jun-Hong Cai, Shu-Ying Yang, Yongchao Ren, Tian Feng, Tianbo Jin, Zhuo-Ri Li

**Affiliations:** 1 Hainan Provincial People’s Hospital, Clinic of Gynecology and Obstetrics, Haikou, China; 2 Hainan Provincial People’s Hospital, Central Laboratory, Haikou, China; 3 Northwest University School of Life Sciences, Shaanxi, China; 4 National Engineering Research Center for Miniaturized Detection Systems, Shaanxi, China

**Keywords:** Polycystic ovary syndrome, DENND1A, rs2479106, rs10818854, meta-analysis

## Abstract

**Objective:** The rs2479106 and rs10818854 polymorphisms in the DENND1A gene have been reported to be extensively associated with risk of polycystic ovary syndrome (PCOS). However, the results from these studies remained inconclusive and conflicting. To detect a true association of rs2479106 and rs10818854 polymorphisms with PCOS risk, a single study may be underpowered, particularly for those studies with inadequate sample size. Therefore, we performed a meta-analysis of all available studies to explore this association.

**Methods:** All studies published up to March 2015 on the association were identified by searching electronic databases PubMed, EMBASE, Web of Science, and China National Knowledge Infrastructure. Studies containing available genotype frequencies of those 2 polymorphisms were chosen, and the odds ratios and associated 95% confidence intervals were calculated using fixed- or random- effects models.

**Results:** A total of 8 studies about s2479106 polymorphism (8185 cases and 28675 controls) and 5 studies about rs10818854 polymorphism (6638 cases and 27443 controls) met the inclusion criteria for the meta-analysis. Overall, significant increase of PCOS risk was found between DENND1A-rs10818854 and PCOS susceptibility. In addition, we also found an increased risk of PCOS in rs2479106 allele model, heterozygote variant genetic model, and dominant genetic model.

**Conclusion:** This meta-analysis suggested that rs2479106 and rs10818854 polymorphisms in the DENND1A gene were associated with increased risk of PCOS. To validate the association between these polymorphisms and PCOS susceptibility, further large and well-designed studies are needed.

## WHAT IS ALREADY KNOWN ON THIS TOPIC?

The rs2479106 and rs10818854 polymorphisms in the DENND1A gene have been reported to be extensively associated with risk of polycystic ovary syndrome (PCOS). However, the results from these studies remained inconclusive and conflicting.

## WHAT THIS STUDY ADDS?

This meta-analysis suggested that the DENND1A gene s2479106 and rs10818854 polymorphisms were associated with increased risk of PCOS.

## INTRODUCTION

Polycystic ovary syndrome (PCOS), known as the most common endocrinopathy in women of reproductive age, is a hyperandrogenic and ovulatory disorder (1). It affects about 5-8% of child-bearing women and is also associated with obesity and several cardiometabolic abnormalities, including metabolic syndrome, insulin resistance (IR), diabetes mellitus type 2, dyslipidemia, atherosclerosis, and hypertension (2). Despite PCOS prevalence and health implications, there is no gold standard for long-term treatment of women with PCOS and the etiology of PCOS remain unclear. Interestingly, it has also been demonstrated that PCOS is a multifactorial disease with polygenic nature, and this heterogeneity complicates the effort to investigate additional genetic components of its pathogenesis (3). The DENN domain containing 1a (DENND1A) gene, a member of a family of 18 human genes termed “connecdenns”, has gained recognition as a strong PCOS susceptibility gene in several studies (4,5).

DENND1A, or connecdenn1, encodes a protein containing domains differentially expressed in normal and neoplastic cells (DENN). The DENND1A protein involves in endosomal membrane trafficking and acts as a guanine exchange factor and interacts with members of the Rab family of small GTPases Rab35 (4,6). DENND1A is ubiquitously expressed and the protein is present in high levels in the brain and kidneys (7). In addition, DENND1A affects a wide range of physiological processes, and it is expected that DENND1A might influence the pathogenesis of PCOS through misregulation of endoplasmic reticulum aminopeptidase1 (ERAP1) (5,8).

Over the last two decades, a number of studies have been conducted to investigate the potential association between DENND1A genomic region and PCOS risk in humans. Among the many polymorphisms of DENND1A genes, rs2479106 and rs10818854 polymorphisms have received much attention. Several studies have previously suggested that the rs2479106 and rs10818854 polymorphisms were associated with an increased risk of PCOS (9). However, other studies have failed to confirm such an association (10,11), presumably due to the relatively small samples of individual studies, possible selective bias, and various genetic backgrounds. Herein, to acquire more comprehensive evidences, we conducted a meta-analysis to assess the effect of the two polymorphisms on the risk of PCOS.

## METHODS

**Search Strategy and Selection Criteria**

We conducted a PubMed search, a Google Scholar search, an EMBASE search, and a China National Knowledge Infrastructure (CNKI) search using the keywords “DENND1A”, “polycystic ovary syndrome”, “rs2479106”, “rs10818854”, and “polymorphism” for the articles. We also supplemented this search by reviewing the reference lists of all retrieved publications. If more than one article was published by the same author using the same case series, we selected the latest research. The relevant search was finished in March 15, 2015. Data on sample characteristics were extracted by 2 independent reviewers who reached a consensus regarding inclusion or exclusion of the article. For the meta-analysis, the following inclusion criteria were considered: 1) Unrelated case-control studies; 2) about rs2479106, rs10818854 polymorphisms and risk of polycystic ovary syndrome; 3) describing useful genotype frequencies; 4) sufficient genotypes data were presented to calculate the odds ratios (ORs); 5) conforming to Hardy-Weinberg (H-W) equilibrium [HWE was tested for genotype frequency distributions of single nucleotide polymorphism (SNP). If there would be deviation from HWE, the results should be interpreted with caution because the observed genotype distribution in control population does not represent genotype distribution in the overall population]; 6) pathological diagnoses and the sources of cases and controls should be clearly described. The exclusion criteria were: 1) Incomplete data; 2) non-case-control studies; 3) duplicated publications; 4) unbalanced matching in patient populations; and 5) lack of approval of local ethics committees.

**Data Extraction**

Two separate investigators reviewed and extracted data from all of the eligible publications independently according to the inclusion and exclusion criteria listed above. The following information will be collected: Characteristics of the methodological research project, the first author’s name, year of publication, country of origin, ethnicity, source of controls, number of cases and controls, PCOS confirmation criteria, genotyping method, genotype frequency for cases and controls, and HWE of controls.

**Statistical Analysis**

The meta-analysis evaluated the overall association between the DENND1A polymorphism and the risk of PCOS using ORs with the corresponding 95% confidence interval (CI) for each study. The significance of the pooled OR was determined by Z test and a p-value of less than 0.05 was considered significant. Different ORs were calculated using the following models: the allele model (A vs. a), the additive genetic model (AA vs. Aa/Aa vs. aa), the dominant genetic mode (AA+Aa vs. aa), the recessive genetic model (AA vs. Aa+aa), heterozygote variant genetic model (Aa vs. aa), and homozygous variant genetic model (AA vs. aa). The heterogeneity of these studies was tested by the χ2 based Q test and I2 statistics (12,13). We considered the result of PQ<0.1 or I2≥50% as indicative of heterogeneity according to Cochrane Handbook, a random-effects (REs) model (the DerSimonian and Laird method) was used to estimate the summary ORs (14); Otherwise, PQ≥0.1 or I2<50%, indicating the absence of heterogeneity, the fixed-effects the (Mantel-Haenszel method model) was used (15). If heterogeneity was presented in this meta-analysis, meta-regression and subgroup analyses were conducted by grouping studies that showed similar characteristics, such as ethnicity, control sources, and genotyping methodology. We also performed meta-analysis using REs models as a sensitivity analysis to examine the robustness of our findings to alternative methods of pooling. If potential publication bias was found in the meta-analyses, contour-enhanced funnel plots and Egger’s test were performed to explore the probable source of publication bias. All statistical analyses were performed using Stata 8.0 (TX, USA) and RevMan 4.2 (Cochrane Collaboration, Oxford, UK).

## RESULTS

**Characteristics of Studies**

**Figure 1:**
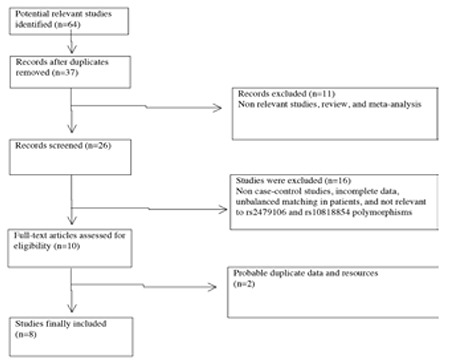
The flow chart of the study selecting process

A total of 64 articles relevant to search keywords were identified based on the search criteria. After removing 27 duplications, 37 articles remained for which the full-text article was retrieved. By reading the titles and abstracts, we excluded 11 articles, including non-relevant studies, reviews, and meta-analysis. After retrieving the full text of the remaining 26 articles, we excluded 16 articles because of the following reasons: non-case-control studies, incomplete data, unbalanced matching in patients, and not relevant to rs2479106 and rs10818854 polymorphisms. Of these 10 articles, the Han Chinese samples (828 participants) in the Zhao et al (16) study might have overlapped subjects used in the Cui et al (17) study, and the Han Chinese groups (GWAS: 744 cases and 895 controls, Replication 1: 2840 cases and 5012 controls, Replication 2: 498 cases and 780 controls) in the Chen et al (8) study were also likely overlapped with those in the Shi et al (9) study. Finally, 8 relevant articles were included in the final meta-analysis (Flow diagram shown in Figure 1).

Overall, a total of 8 case-control studies about rs2479106 with 8185 cases and 28675 controls were included. Simultaneously, 5 studies about rs10818854 with 6638 cases and 27443 controls were analyzed. The main characteristics of these studies are summarized in Table 1. These studies focused on different populations of various ethnicities: 2 studies of Chinese, 1 study of Bahrain, 1 study of European, 1 study of Denmark, 1 study of Austria, and 2 studies of the USA. The distribution of the genotypes in the control subjects was consistent with HWE.

**Meta-analysis Results**

The determined association between rs2479106 polymorphism and the risk of PCOS are shown in Table 2 and Supplementary 1A-1B. Overall, individuals carrying the rs2479106 G allele had significantly increased risk for PCOS compared to those carrying A allele (OR=1.202, 95% CI: 1.070-1.350, p=0.002) in the allele model. This was also the case for GA vs. AA in the heterozygote variant genetic model (OR=1.266, 95% CI: 1.140-1.407, p<0.001) and for GG/GA vs. AA in the dominant genetic model (OR=1.277, 95% CI: 1.155-1.411, p<0.001). In the stratified analysis by ethnicity, significantly elevated PCOS risk was found in Asians when all studies were pooled into the meta-analysis (G vs. A: OR=1.301, 95% CI: 1.219-1.389, p<0.001; GG vs. AA: OR=1.559, 95% CI: 1.234-1.970, p<0.001; recessive genetic model: OR=1.417, 95% CI: 1.127-1.782, p=0.003; additive genetic model: OR=1.264, 95% CI: 1.156-1.382, p<0.001). However, no increased risk was detected in Caucasians in the allele model, the homozygote contrast, the recessive model, or the additive model (all p>0.05). Further subgroup analysis by ethnicity was not done due to the small number of studies.

The meta-analysis findings of the correlation between rs10818854 and PCOS risk are summarized in Table 3 and Supplementary 2. A significant association was found between the rs10818854 A allele and PCOS risk in the allele model (OR=1.395, 95% CI: 1.148-1.694, p=0.001). Similarly, this association was found in the additive genetic model (OR=1.543, 95% CI: 1.381-1.725, p<0.001). In the subgroup analysis of ethnicity, rs10818854 A was significantly associated with an increased risk of PCOS in Caucasians or Asians in the allele model (OR=1.530, 95% CI: 1.170-2.000, p=0.002; OR=1.362, 95% CI: 1.073-1.729, p=0.011), potentially suggesting that the A variant might exhibit a higher risk of PCOS between different ethnical populations.

**Tests of Heterogeneity**

We reckoned the heterogeneity between each of the studies using the Q-test. For rs2479106-related PCOS risk (Table 2), significant heterogeneity between studies was observed in some comparisons (G vs. A: PQ=0.002; GG vs. AA: PQ=0.016; recessive genetic model: PQ=0.011; additive genetic model: PQ=0.038). Then, the REs models were used to evaluate the combined ORs when necessary. Vice versa, the other models used fixed-effects model to analyze the ORs. After stratifying by ethnicity, no heterogeneity was found between studies (PQ>0.05). For rs10818854 (Table 3), there was significant heterogeneity between studies under the allele model (G vs. A: PQ=0.004), and ORs were pooled under REs model. Subsequently, we performed subgroup analyses stratified by ethnicity. However, heterogeneity still existed among Asians (G vs. A: PQ=0.002).

**Sensitivity Analysis**

Since significant heterogeneity across studies was observed for some models, we performed a sensitivity analysis to assess the influence of each individual study on the pooled OR by sequentially removing the individual study (statistics of study removal). The results suggested that no single study dramatically change the pooled ORs, indicating that our results were robust and reliable (Figures Supplementary 1 and Supplementary 2).

**Publication Bias**

The results of Egger’s test did not show any evidence of publication bias in any comparison model (p>0.05).

## DISCUSSION

The DENND1A gene has been mapped to chromosome 9q22.32 and consists of 22 exons extending over 500,000 bases (4). Rs2479106 and rs10818854 are located in intron regions of the DENND1A gene and encode the protein DENND1A, and as such, SNPs located within DENND1A introns may influence DENND1A expression by interacting with upstream or downstream chromosomal regions (18). Expression of this gene has been reported in testes, ovarian theca cells, and H295 adrenal carcinoma cells (19).

To date, a number of epidemiological studies have evaluated the association between DENND1A rs2479106 and rs10818854 polymorphisms and PCOS risk, but the results remain inconclusive. Meta-analysis can comprehensively evaluate and quantitatively analyze multiple research results from a critical and objective perspective, thereby improving the efficiency of statistical analysis and tests.

In this study, we found a significant association between the variant rs10818854 and increased risk of PCOS. Nevertheless, the results were conflicting in previous studies. Shi et al (9) found a correlation between rs10818854 variant allele and elevated PCOS risk in genome-wide association study (GWAS 1), and Cui et al (17), Goodarzi et al (11) and Welt et al (20) also identified an increased risk. Contrary to these results, Shi et al (9) GWAS 2 and Gammoh et al (21) did not detect an association between rs10818854 variant allele and PCOS risk. In addition, in the subgroup analysis by ethnicity, the association between rs10818854 variant allele and PCOS risk was also found in Asians and Caucasians. Taken together, it may be concluded that DENND1A rs10818854 polymorphism was associated with PCOS risk in common population. Although we herein confirmed the association between the rs10818854 polymorphism and PCOS risk, whether this SNP is causative is still uncertain. Significantly, previous study have shown rs10818854 is in high linkage disequilibrium with rs10986105 in European populations (r2=0.83 in HapMap CEU) (11). Thus, a systematically functional validation study is necessary to clarify the role of the rs10818854 polymorphism in the development of PCOS risk.

Reviewing the past studies about DENND1A-rs2479106 polymorphism and the risk of PCOS, Shi et al (9) and Cui et al (17) found an increased risk for PCOS associated with rs2479106 variant allele, Lerchbaum et al (22) and Eriksen et al (18) identified a decreased risk, and the other studies did not detect an association between rs2479106 variant allele and PCOS risk (20,21,23). In this study, we analyzed the data from eight available case-control studies. We also found that the rs2479106 polymorphism was associated with elevated PCOS risk in allele model (CC vs. CG), heterozygote variant genetic model (GA vs. AA), and dominant genetic model (GG/GA vs. AA). After stratifying by ethnicity, significantly elevated risk associated with the rs2479106 G allele was only found for PCOS risk among Asians but not in Caucasians in all genetic models. This suggested that DENND1A-rs2479106 may have different effects in different populations. The possible explanation for these discrepancies could be different genetic backgrounds. However, the number of studies in this subgroup was a little small (n=4), which may have insufficient statistical power to detect a slight effect or may reduce the reliability of the results. Notably, the results should be interpreted with caution.

Heterogeneity is a potential problem when interpreting the results of a meta-analysis, and the obvious evidence of between-study heterogeneity in this meta-analysis should be issued. In the present meta-analysis, there were modest heterogeneities in some comparisons for DENND1A-rs2479106 polymorphism and PCOS risk. After stratifying by ethnicity, the subgroup did not show heterogeneity anymore, reflecting that ethnicity might influence the heterogeneities. Nevertheless, for rs10818854-related PCOS risk, there were still notable heterogeneities in Asians in subgroup analyses, which may be caused by the different ethnicities, study quality, genotyping methods, and experimental designs.

There are several limitations in the present study. First, the sample size of our case-control study was relatively small. Some non-English articles, unpublished reports were not included in our meta-analysis, which may bias our results. Second, our meta-analysis was based on unadjusted estimates, whereas a more precise analysis of the various groups should be conducted according to potentially confounding factors, such as age, gender, obesity, drinking and smoking status, menopausal status, use of contraceptives, environment factors, and so on.

## CONCLUSION

In summary, this meta-analysis suggests that the rs10818854 polymorphism may be associated with increased risk of PCOS in the allele model and additive model. In addition, increased risk of PCOS was found in rs2479106 allele mode, the heterozygote variant genetic model, and dominant genetic model. Our findings comprehensively evaluate the association between DENND1A SNPs and PCOS risk and provide the basis for subsequent research of molecular mechanisms underlying the identified association. Further large sample size and well-designed studies in different ethnic populations are needed to verify our observations.

**Ethics**

Ethics Committee Approval: Human Research Committee of Hainan Provincial People’s Hospital for Approval of Research Involving Human Subjects, Informed Consent: Each participant gave written informed consent.

Peer-review: External and Internal peer-reviewed.

## AUTHORSHIP CONTRIBUTIONS

Surgical and Medical Practices: Shan Bao, Jun-Hong Cai, Concept: Shan Bao, Jun-Hong Cai, Shu-Ying Yang, Design: Tianbo Jin, Zhuo-Ri Li, Data Collection or Processing: Yongchao Ren, Tian Feng, Analysis or Interpretation: Yongchao Ren, Tian Feng, Literature Search: Yongchao Ren, Tianbo Jin, Writing: Yongchao Ren.

Financial Disclosure: This work was supported by social development of Hainan province special fund of science and technology (SF201302).

## Figures and Tables

**Supplementary 1a:**
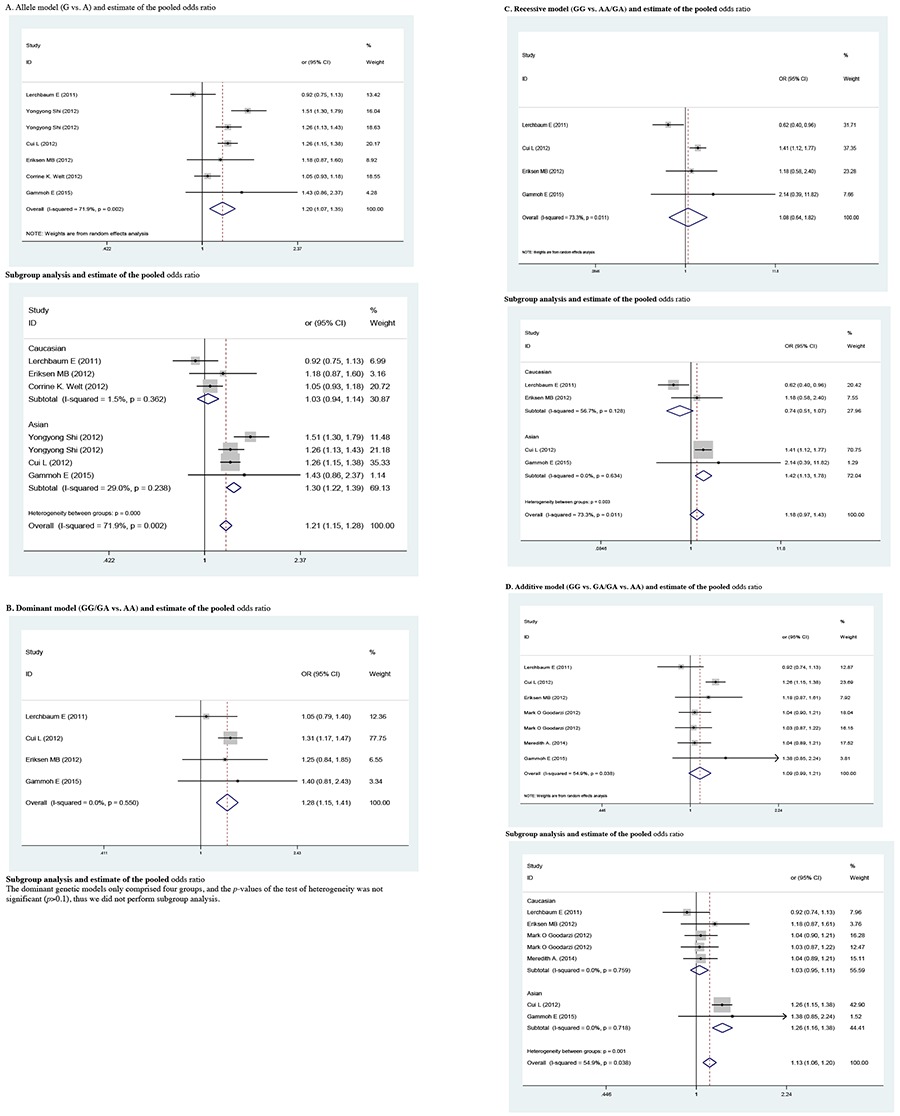
Pooled odds ratio with 95% confidence interval for the association between rs2479106 and Polycystic ovary syndrome risk in all genetic model comparisons (risk allele: G)

**Supplementary 1b:**
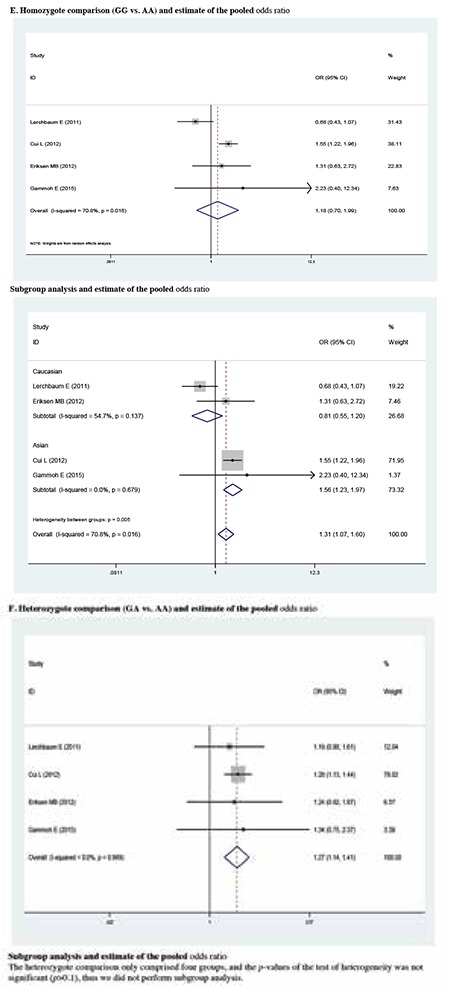
Pooled odds ratio with 95% confidence interval for the association between rs2479106 and Polycystic ovary syndrome risk in all genetic model comparisons (risk allele: G)

**Supplementary 2:**
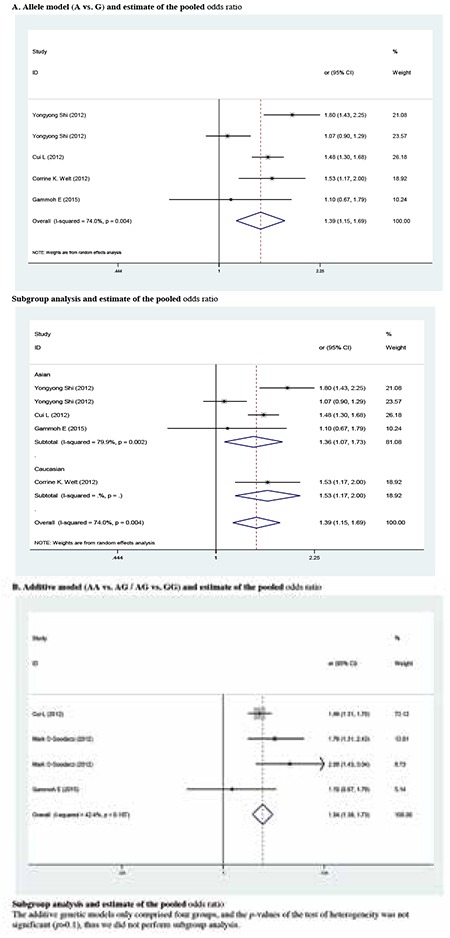
Pooled odds ratio with 95% confidence interval for the association between rs10818854 and Polycystic ovary syndrome risk in genetic model comparisons (risk allele: A)

**Table 1:**
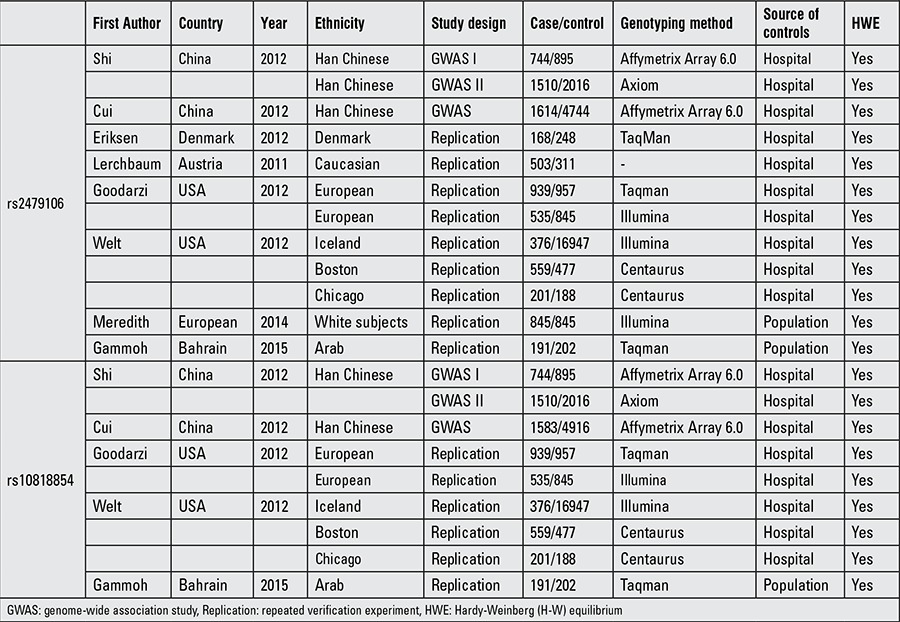
Characteristics of studies included in the meta-analysis

**Table 2:**
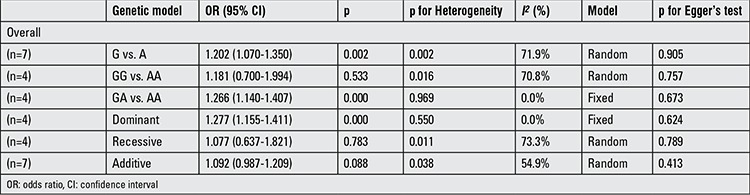
Pooled odds ratio with 95% confidence interval for the association between rs2479106 and Polycystic ovary syndrome risk in the meta-analysis

**Table 3:**
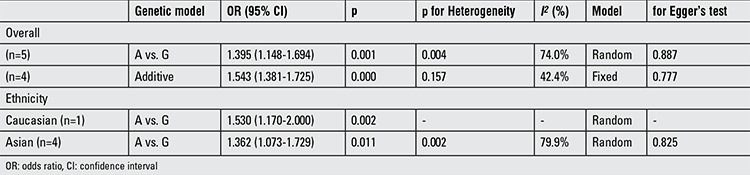
Pooled odds ratio with 95% confidence interval for the association between rs10818854 and Polycystic ovary syndrome risk in the meta-analysis

**Figure Supplementary 1:**
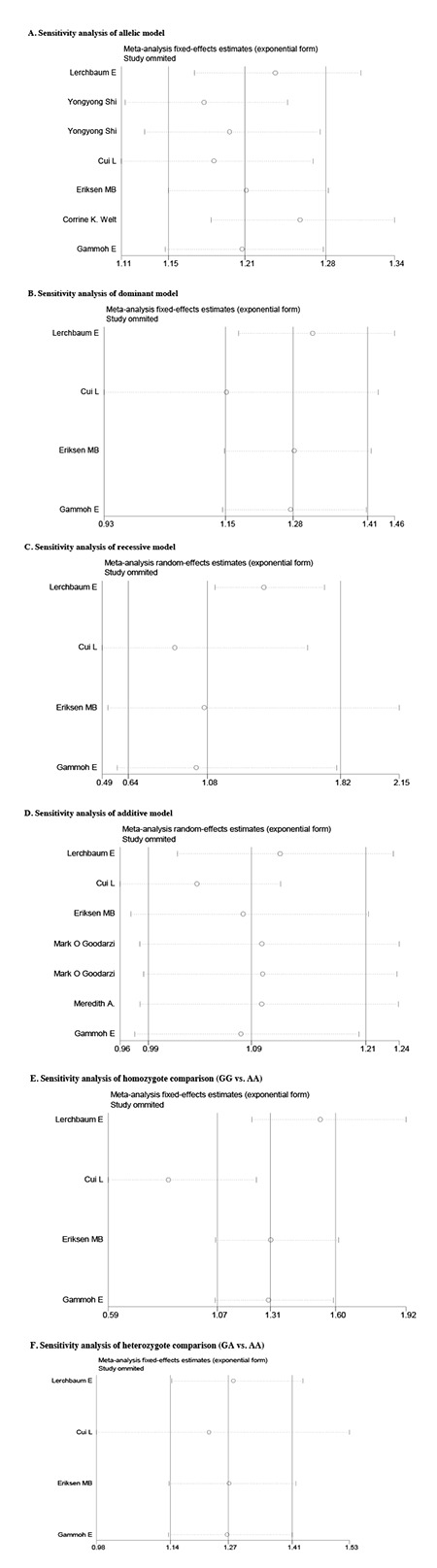
Sensitivity analysis of rs2479106 polymorphism. The analysis was performed by omitting each study in turn. The two ends of the dotted lines represent the 95% confidence interval

**Figure Supplementary 2:**
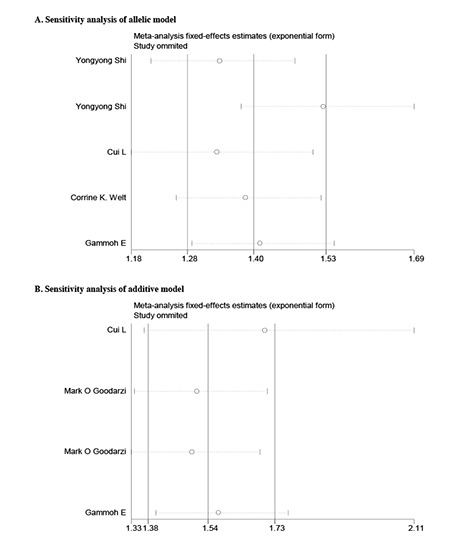
Sensitivity analysis of rs10818854 polymorphism. The analysis was performed by omitting each study in turn. The two ends of the dotted lines represent the 95% confidence interval
